# Clinical parameters and biological markers associated with acute severe ocular complications in Stevens-Johnson syndrome and toxic epidermal necrolysis

**DOI:** 10.1038/s41598-021-99370-1

**Published:** 2021-10-12

**Authors:** Rawiphan Panpruk, Vilavun Puangsricharern, Jettanong Klaewsongkram, Pawinee Rerknimitr, Thanachaporn Kittipibul, Yuda Chongpison, Supranee Buranapraditkun

**Affiliations:** 1 Cornea and Refractive Surgery Unit, Department of Ophthalmology, Faculty of Medicine, Chulalongkorn University, Bangkok, Thailand; 2 Excellence Center of Cornea and Limbal Stem Cell Transplantation, Department of Ophthalmology, 1873 King Chulalongkorn Memorial Hospital, Faculty of Medicine, Chulalongkorn University, Pathumwan, Bangkok, 10330 Thailand; 3 Division of Allergy and Clinical Immunology, Department of Medicine, Faculty of Medicine, Chulalongkorn University, Bangkok, Thailand; 4 Division of Dermatology, Department of Medicine, Faculty of Medicine, Chulalongkorn University, Bangkok, Thailand; 5 The Skin and Allergy Research Unit, Chulalongkorn University, Bangkok, Thailand; 6 King Chulalongkorn Memorial Hospital, Thai Red Cross Society, Bangkok, Thailand; 7 Center of Excellence in Biostatistics, Research Affairs, Faculty of Medicine, Chulalongkorn University, Bangkok, Thailand; 8 Center of Excellence in Vaccine Research and Development (Chula Vaccine Research Center, Chula VRC), Faculty of Medicine, Chulalongkorn University, Bangkok, 10330 Thailand; 9 Thai Pediatric Gastroenterology, Hepatology and Immunology (TPGHAI) Research Unit, Faculty of Medicine, Chulalongkorn University, Bangkok, 10330 Thailand

**Keywords:** Conjunctival diseases, Corneal diseases, Skin diseases, Risk factors, Immunological disorders, Predictive markers

## Abstract

Stevens-Johnson syndrome (SJS) and toxic epidermal necrolysis (TEN) are severe cutaneous adverse drug reactions with high mortality rates. Its sequelae, such as blindness, persist even after recovery. Patients with SJS/TEN should be accurately diagnosed and receive appropriate treatment as soon as possible. Therefore, identifying the factors for severity prediction is necessary. We aimed to clarify the clinical parameters and biological markers that can predict acute severe ocular complications (SOCs) in SJS/TEN. This retrospective cross-sectional study enrolled 47 patients with SJS/TEN who were divided into two groups according to ocular severity at acute onset: non-severe ocular complications group (n = 27) and severe ocular complications group (n = 20). Multivariate logistic regression analysis revealed that disease severity (body surface area detachment ≥ 10%) was a predictive factor for acute SOCs, and older age (≥ 60 years) was marginally significantly predictive of SOCs. Serum biomarker levels of S100A8/A9 and granulysin were marginally significant and tended to increase in the SOC group. Therefore, during the early acute stage, focusing on disease severity, patient age, and serum inflammatory biomarkers (S100A8/A9 and granulysin) might help predict SOC progression in patients with SJS/TEN who need prompt and aggressive ocular management to prevent severe ocular sequelae.

## Introduction

Stevens-Johnson syndrome (SJS) and toxic epidermal necrolysis (TEN) are severe cutaneous adverse reactions (SCARs) characterized by blistering mucocutaneous lesions leading to necrosis and sloughing of the skin epidermis and mucosa, mostly including ocular, oral, and genital membranes. SJS and TEN (SJS/TEN) can be considered as a spectrum of increasing severity and mortality and are distinguished based on the involvement of the body surface area (BSA). SJS is a less severe form, with skin sloughing of less than 10% of BSA, whereas TEN is more severe, with sloughing of more than 30% of BSA. When the involvement is greater than 10% but less than 30% of the body surface, it is referred to as the SJS/TEN overlap syndrome^1^. The incidence of SJS and TEN is approximately 0.4 to 1 case per 1 million persons and 1 to 6 cases per 1 million persons, respectively^2^. Although rare, SJS/TEN is significant because of its high mortality and morbidity rates^3,4^.

The score of toxic epidermal necrolysis (SCORTEN), is a standard scoring system for assessing SJS/TEN severity^3^. Several clinical parameters, including age, malignancy, tachycardia, initial BSA of epidermal detachment, serum urea, serum glucose, and bicarbonate, have been used for evaluation. Other predictors of mortality in SJS/TEN are age, pre-existing comorbidities, hematological malignancy, septicemia, pneumonia, tuberculosis, and renal failure^5^. Among the various etiological factors for SJS/TEN, drug reactions are the most common; allopurinol, antibiotics, antipsychotics, nonsteroidal anti-inflammatory drugs (NSAIDs), and antiepileptic medications are the most frequently associated^2,3^. Other causes such as infection from herpes simplex virus, mycoplasma, and other unidentified etiologies may be responsible for up to 15% of cases^6^.

Ocular complications are the major, severe, long-term sequelae following SJS/TEN. They do not always correlate with the severity of the systemic disease^7^. Acute ocular complications develop in 43–81% of patients hospitalized for SJS/TEN. Clinical features range from self-limited conjunctival hyperemia to extensive sloughing of the ocular surface and lid margins^2^. Chronic ocular sequelae occur in 35%–63% of cases^8,9^. Previous studies have shown that acute severe ocular complications (SOCs) are strong predictors of chronic ocular sequelae^10–14^. Delayed or inadequate ocular treatment in the acute stage can result in irreversible chronic SOCs, such as severe dry eye, trichiasis, conjunctival invasion into the cornea, and conjunctival scarring, resulting in visual disturbances and substantially lower overall health-related quality of life^15^. Treatments for chronic SOCs remain challenging and are prone to failure, thereby emphasizing the importance of intensive management of SOCs in the acute phase to prevent chronic SOCs.

Currently, SJS/TEN is considered a delayed T cell-mediated hypersensitivity reaction. The pathological mechanisms underlying SJS/TEN are not well understood. Cytotoxic T lymphocytes (CD8 + T cells) and natural killer (NK) cell immune-mediated reactions are the major immunologic component^5,16,17^. This immune-mediated cytotoxic reaction against keratinocytes leads to massive apoptosis of the keratinocytes and/or necroptosis and causes an acute inflammatory vesiculobullous reaction of the skin^18^. In the eye, the early vacuolation of basal keratinocytes with the absence of a lymphocytic infiltrate offers inflammatory cytokines an important role in the pathogenesis of acute ocular inflammation^19^.

A severe cytokine storm occurs on the ocular surface during the SJS/TEN onset^20^. Several biomarkers, such as IL-6, IL-8, IL-15, IL-10, IL-13, IL-17, TNF-α, IFN-γ, and granulysin (GYLN), are increased in the skin lesions, peripheral blood mononuclear cells, and plasma of patients with SJS/TEN^20–26^. Some of them, such as GYLN and IL-15, can reflect the severity of SJS/TEN^23,26^, but their association with ocular involvement has not been widely explored.

Most previous studies have focused on predicting factors for chronic SOCs in SJS/TEN. In contrast, there have been very few studies on the prognostic value of acute SOCs^27,28^. Moreover, none of the studies have evaluated the correlation between biological markers and SOCs. The purpose of this study was to determine the clinical parameters and serum biomarkers that can predict acute SOCs in patients with SJS/TEN. Prompt identification of the high-risk group will guide clinicians in early intervention and thereby prevent long-term devastating ocular conditions.

## Results

### Patient characteristics and causative drugs of SJS/TEN

The patient characteristics are summarized in Table 1. From a total of 47 SJS/TEN cases involved in the study, 35 (74.47%) were classified as SJS, six (12.77%) as SJS/TEN overlap, and six (12.77%) as TEN. Forty-three of the 47 patients (91.49%) presented with ocular involvement in the acute phase. The acute ocular severity score (Table 2) was grade 0 in four cases (8.51%), grade 1 in 11 cases (23.40%), grade 2 in 12 cases (25.53%), and grade 3 in 20 cases (42.55%). We classified patients with acute ocular complications into two groups: non-severe and severe. Twenty-seven patients with ocular severity grades 0–2 were included in the non-severe group, and the remaining 20 patients were included in the severe group (ocular severity grade 3). The mean SCORTEN score (mean ± SD) was 1.80 ± 1.27. Considering the SCORTEN score, the SOC group showed higher scores than the non-severe group (2.25 ± 1.37 vs. 1.44 ± 1.08). The mean initial visual acuity at the onset of SJS/TEN (logMAR_ ± SD) was comparable in both groups (0.37 ± 0.53 and 0.46 ± 0.57 in the non-severe group and severe group, respectively).

**Table 1:** Analysis of the correlations between patient characteristics (demographic and clinical parameters) and acute severe ocular complications in SJS/TEN patients.

Characteristics	Non-severe ocular involvement n (%)	Severe ocular involvement n (%)	Univariate^‡^ OR (95% CI)	Multivariate^‡^ OR (95% CI)
**Diagnosis**				
BSA detachment ≥ 10%	4 (14.28)	8 (40)	6.00 (1.35–26.72)**	5.05 (1.02–24.94)**
BSA detachment < 10%	23 (85.19)	12 (60)	1	1
**Age**				
≥ 60 years	4 (14.81)	7 (35)	3.95 (0.87–17.99)*	3.88 (0.75–19.98)
< 60 years	23 (85.19)	13 (65)	1	1
**Gender**				
Female	16 (59.26)	10 (50)	0.80 (0.24–2.56)	
Male	11 (40.74)	10 (50)	1	
**SCORTEN**				
3–6	4 (16)	9 (45)	4.3 (1.08–17.17)**	2.82 (0.62–12.96)
0–2	21 (84)	11 (55)	1	1
**Initial VA (logMAR)**				
≥ 1	1 (4.6)	3 (17.64)	1.35 (0.42–4.31)	
< 1	21 (95.4)	14 (82.36)	1	
**Number of mucosal involvements**^†^				
3–4	17 (62.96)	13 (65)	1.05 (0.31–3.57)	
0–2	10 (37.04)	7 (35)	1	
**Flu-liked symptom**				
Yes	5 (18.51)	7 (35)	2.15 (0.56–8.25)	
No	22 (81.48)	13 (65)	1	
**HIV infection**				
Yes	8 (29.63)	6 (30)	0.91 (0.25–3.25)	
No	19 (70.37)	14 (70)	1	
**Autoimmune disease**				
Yes	4 (14.81)	2 (10)	0.64 (0.10–3.89)	
No	23 (85.19)	18 (90)	1	
**Malignancy**				
Yes	4 (14.81)	4 (20)	1.44 (0.3–6.61)	
No	23 (85.19)	16 (80)	1	
**Sepsis**				
Yes	3 (11.11)	4 (20)	1.83 (0.36–9.35)	
No	24 (88.89)	16 (80)	1	
**Antibiotics**^**††**^				
Yes	14 (51.85)	4 (20)	0.23 (0.06–0.88)**	
No	13 (48.15)	16 (80)	1	
**Anticonvulsant**^**††**^				
Yes	8 (29.63)	4 (20)	0.6 (0.11–2.77)	
No	19 (70.37)	16 (80)	1	
**Allopurinol**^**††**^				
Yes	4 (14.81)	5 (25)	1.89 (0.34–11.19)	
No	23 (85.19)	15 (75)	1	
**NSAIDs or cold remedies**^**††**^				
Yes	2 (7.41)	1 (5)	0.61 (0.05–7.2)	
No	25 (92.59)	19 (95)	1	

*BSA* body surface area, *SJS* Stevens–Johnson syndrome; *TEN* toxic epidermal necrolysis; *SCORTEN* Severity-of-Illness Score for toxic epidermal necrolysis; *VA* visual acuity; *SD* standard deviation; *LogMAR* logarithmic minimum angle of resolution; *HIV* human immunodeficiency virus; *NSAIDs* nonsteroidal anti-inflammatory drugs; *DMARDs* disease-modifying antirheumatic drugs.

^†^Oral mucosa, ocular mucosa, genital mucosa and other mucosa.

^††^Causative drug.

^**‡**^n = 45.

^∗^*P* < 0.1; **P < 0.05.

**Table 2:** Acute ocular severity grading of Stevens–Johnson syndrome and toxic epidermal necrolysis^27^.

Acute ocular manifestations	Grade	Number of patients (%)
No ocular involvement	0	4 (8.51)
Conjunctival hyperemia	1	11 (23.40)
Either ocular surface epithelial defect or pseudomembrane formation	2	12 (25.53)
Both ocular surface epithelial defect and pseudomembrane formation	3	20 (42.55)

Among all causative drugs (Table 3), antibiotics were the most common cause (35.84%), followed by anticonvulsants (22.64%) and allopurinol (17.00%). Thirty-five patients (74.46%) had underlying diseases, including human immunodeficiency virus (HIV) infection (14 cases; 22.79%), autoimmune disease (6 cases; 12.76%), and malignancy (8 cases; 17.02%). Thirteen out of 14 patients with HIV infection (92.85%) had a CD4 count of less than 200 cells/μl (mean, 116.79 ± 87.096; range 12–688).

**Table 3:** Causative drugs of Stevens–Johnson syndrome or toxic epidermal necrolysis.

Causative drugs	Number of culprit drugs*	Percentage (%)
**Antibiotics**	19	35.84
Trimethoprim-Sulfamethoxazole	6	
Beta lactam	4	
Quinolones	2	
Isoniazid	3	
Rifampicin	3	
Dapsone	1	
**Anticonvulsants**	12	22.64
Phenytoin	6	
Carbamazepine	3	
Lamotrigine	3	
**Allopurinol**	9	17.00
**Antifungal**	4	7.55
**Antiviral**	2	3.77
**NSAIDs**	2	3.77
**Cold remedies**	1	1.89
**Others**	4	7.54
DMARDs	1	
Antiasthma (Doxofylline)	1	
Oral whitening pill containing gluthathione	1	
Cancer Immunotherapy (Atezolizumab)	1	

*NSAIDs* nonsteroidal anti-inflammatory drugs; *DMARDs* disease-modifying antirheumatic drugs.

*Include both single possible culprit drug and more than one possible culprit drugs.

### Clinical factors related to acute SOC in SJS/TEN

Clinical factors related to acute SOCs are listed in Table 1. A total of 47 patients were included in the analysis. Univariate logistic regression analysis showed that BSA detachment ≥ 10% (OR = 6.0, 95% CI 1.35–26.72, *P* = 0.019), age ≥ 60 years (OR = 3.95, 95% CI 0.87–17.99, *P* = 0.076), SCORTEN ≥ 3 (OR = 4.30, 95% CI 1.08–17.17, *P* = 0.039), and antibiotics (OR = 0.23, 95% CI 0.06–0.89, *P* = 0.033) were associated with SOCs (ocular severity grade 0–2 vs grade 3) as the candidate predictive factors. Multivariate logistic regression analysis revealed that BSA was a significant predictive factor for acute SOCs (adjusted OR: 5.05, 95% CI 1.02–24.94, *P* = 0.047). However, we found that age ≥ 60 years was a marginally significant predictive factor (adjusted OR: 3.88, 95% CI 0.75–19.98, *P* = 0.105).

### Analysis of serum biomarkers between patients with SJS/TEN and healthy controls

**Figure 1:**
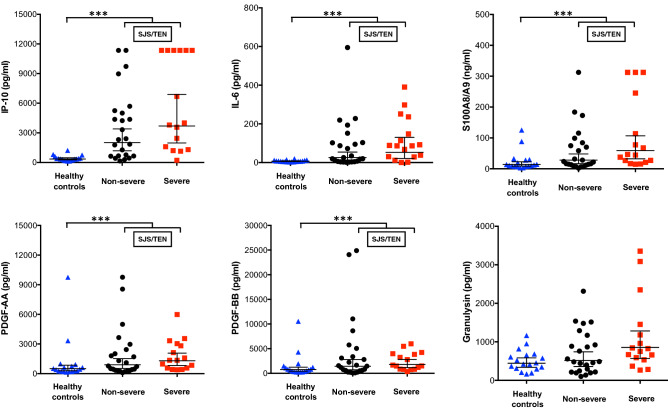
Scatter plot graphs of serum biomarker levels (geometric means) in healthy controls, and those from patients with SJS/TEN with non-severe and severe ocular complications. Representative scatter plot graphs of IP-10, IL-6, S100A8/A9, PDG-AA, PDG-BB, and granulysin levels. The geometric means with the 95% confidence interval of each biomarker in healthy controls (HC), SJS/TEN with non-severe ocular complications, and SJS/TEN with severe ocular complications group are shown. The Mann–Whitney test was used to compare biomarker levels between the HC and SJS/TEN groups and between the non-severe and severe ocular complications groups. IP-10, IL-6, S100A8/A9, PDG-AA, and PDG-BB were significantly higher in SJS/TEN group than in HC. When comparing the non-severe and severe ocular complication groups, any biomarkers showed significant differences. S100A8/A9 and granulysin were marginally significant and tended to increase in the severe ocular complications group. *IP-10* interferon-γ-inducible protein 10; *IL-6* interleukin 6; *S100A8/A9* heterodimeric of S100 calcium binding protein A8 and S100 calcium binding protein A9; *PDG-AA* PLATELET-derived growth factor AA; *PDGF-BB* platelet-derived growth factor BB*.* *P < 0.05, **P < 0.01, ***P < 0.001.

The geometric means and the geometric mean ratios (GMR) of serum biomarker levels between healthy controls (HC) and patients with SJS/TEN with different ocular severities are shown in Table 4 and Fig. 1. After excluding seven patients with sepsis to rule out possible confounders, we compared biomarker levels in the serum of patients with SJS/TEN (N = 40) with HC (N = 18).

**Table 4:** The comparison of serum biomarkers levels between healthy controls and SJS/TEN patients with different ocular severity.

	(A)				(B)			
	Comparison of biomarkers between Healthy controls group and SJS/TEN group				Comparison of biomarkers between non-severe ocular complications group and severe ocular complications group			
Biomarkers	Healthy controls	SJS/TEN	GMR^†^	95% CI	SJS/TEN		GMR^††^	95% CI
					Non-severe	Severe		
	GM, ng/ml (%CV)	GM, ng/ml (%CV)			GM, ng/ml (%CV)	GM, ng/ml (%CV)		
IP-10	356.2	2562.6	7.2	(3.89–13.30)***	2008.7	3692.6	1.8	(0.83–4.07)
	(63.4)	(190.3)			(192.8)	(170.7)		
IL-6	6.9	33.0	4.8	(2.09–11.09)***	26.2	46.6	1.8	(0.56–5.49)
	(53.2)	(433.1)			(425.6)	(442.0)		
IL-17A	1.6	0.7	0.4	(0.25–0.65)***	0.8	0.5	0.7	(0.44–1.12)
	(149.0)	(844.1)			(86.6)	(75.6)		
S100A8/A9	14.1	37.9	2.7	(1.38–5.23)**	28.2	58.9	2.1	(0.95–4.61)
	(125.8)	(194.3)			(199.9)	(157.9)		
SCF	111.6	74.3	0.7	(0.51–0.86)**	70.7	79.9	1.1	(0.82–1.56)
	(32.4)	(51.9)			(63.3)	(33.6)		
GYLN	444.5	631.3	1.4	(0.92–2.20)	515.7	855.0	1.7	(0.97–2.84)
	(58.9)	(103)			(104.67)	(88.8)		
CRP	50.2	64.4	1.3	(1.02–1.62)*	63.6	65.8	1.0	(0.80–1.34)
	(47.3)	(41.0)			(42.2)	(40.5)		
OPN	69.1	101.9	1.5	(1.02–2.13)*	89.9	123.0	1.4	(0.85–2.19)
	(39.0)	(84.0)			(97.6)	(59.1)		
ICAM-1	520.7	725.7	1.4	(1.06–1.84)*	683.7	793.6	1.2	(0.83–1.63)
	(43.5)	(55.6)			(58.3)	(51.6)		
PDGF-AA	501.8	1042.8	2.1	(1.11–3.89)*	899.8	1303.0	1.5	(0.7–3.01)
	(145.8)	(158.1)			(194.9)	(107.1)		
PDGF-BB	746.9	1562.3	2.1	(1.03–4.27)*	1420.3	1802.3	1.3	(0.53–3.07)
	(139.0)	(223.8)			(344.1)	(101.9)		
MCP-1	252.4	156.7	0.6	(0.43–0.91)*	145.6	175.0	1.2	(0.73–1.98)
	(38.7)	(88.6)			(76.6)	(108.4)		

(A) Comparison between healthy controls group and SJS/TEN group.

(B) Subgroup comparison between non-severe and severe ocular complications group.

*GM* geometric mean; *GMR* geometric mean ratios; *%CV* percent coefficient of variation; *IP-10* interferon-γ-inducible protein 10; *IL-6* interleukin 6; *S100A8/A9* heterodimeric of S100 calcium binding protein A8 and S100 calcium binding protein A9; *SCF* stem cell factor; *GYLN* granulysin; *CRP* C-reactive protein; *OPN* Osteopontin; *ICAM 1* intercellular adhesion molecule 1; *PDGF AA* platelet-derived growth factor AA*; PDGF BB* platelet-derived growth factor BB; *MCP* monocyte chemotactic protein 1.

^†^GMR in column A = the quotient of SJS/TEN and Healthy controls geometric means.

^††^GMR in column B = the quotient of SJS/TEN with severe ocular complications and SJS/TEN with non-severe ocular complications geometric means.

*P < 0.05, **P < 0.01, ***P < 0.001.

Compared to the serum from the HC group, the SJS/TEN group serum showed significant upregulation of 11 factors. Three biomarkers had *P* values < 0.001, including IP-10, IL-6, and IL-17A. Two biomarkers (SCF and S100A8/A9) had *P* values < 0.01, and five biomarkers (MCP-1, ICAM-1, PDGF-AA, CRP, OPN, and PDGF-BB) had *P* values < 0.05.

Five of the 11 biomarkers had geometric mean ratios (GMR) > 2. The geometric mean of the IP-10 serum concentration in the SJS/TEN group was 7.2 times higher than that in the HC group (GMR = 7.2, 95% CI 3.89–13.30), followed by IL-6, S100A8/A9, PDGF-AA, and PDGF-BB (GMR = 4.8, 95% CI 2.09–11.09; GMR = 2.7, 95% CI 1.38–5.23; GMR = 2.1, 95% CI 1.11–3.89, and GMR = 2.1, 95%CI 1.03–4.27, respectively).

### Subgroup analysis of serum biomarkers between non-severe and SOC in SJS/TEN

We have analyzed different groups of SJS/TEN to compare both non-severe (n = 24) and severe (n = 16) ocular complications (Table 4 and Fig. 1). Although there was no statistically significant difference between the two groups, many cytokines were upregulated in the severe ocular involvement group. Among the biomarkers that were upregulated in the severe group, S100A8/A9 (*P* = 0.067, 95% CI 0.95–4.61) and GYLN (*P* = 0.065, 95% CI 0.97–2.84) were marginally significant and tended to increase in the severe groups.

## Discussion

Acute SOCs in SJS/TEN often lead to chronic SOCs^10–13^. A recent study from Taiwan found a positive correlation between acute SOCs and chronic SOCs grading score among SJS/TEN patients^14^. Nonetheless, the clinical parameters and biological markers associated with acute SOCs remain unclear. Sotozono et al. demonstrated that patient age, NSAIDs, and remedies for the common cold are related to SOCs^27^. Saka et al. identified an association of sulfadoxine with moderate or severe acute ocular involvement in SJS/TEN^28^. Sadek et al. reported that IL-13 and GYLN might play a role in the pathogenesis of ocular complications in SJS/TEN by finding that the corneal epithelium induced by TNF-α can produce IL-13 and GYLN in a dose–response relationship^25^.

From the univariate analysis, our results revealed that BSA detachment ≥ 10%, older age (≥ 60 years), antibiotics as causative drugs, and heightened SCORTEN were associated with acute SOCs. Among these factors, only BSA (detachment ≥ 10%) was identified as a predictive factor for acute SOCs in the multivariate analysis. Yip^29^ and Sotozono et al.^27^ reported no association between BSA detachment and SOC. Interestingly, Yip et al. used the old ocular severity grading, combining both acute and chronic ocular findings into the same analysis, which might not represent the acute phase in SJS/TEN^30^. In contrast, our study uses the severity score, which includes only ocular findings in the acute phase^27^. Furthermore, our study included cases with AOS grade 3 in the SOCs group compared to the study by Sotozono et al.^27^, where patients with both AOC grades 2 and 3 were enrolled in the SOC group, leading us to find a correlation between the disease severity and acute SOCs in this study. This association could be due to similar mechanisms of apoptosis occurring in the skin and eyes^29,31,32^. Further, our data show that SOCs are in line with skin involvement. Thus, patients with SJS/TEN having severe skin involvement should receive intensive ocular treatment for anticipated severe symptoms.

Previous studies have shown that younger age (< 50 years) is associated with acute SOCs^27,33^. They hypothesized that not only younger age but also medication for common cold and viral infections causing cold-like symptoms play an essential role in the development of SOCs^27,33^. In contrast, we found that older age and antibiotic use were associated with acute SOCs in our study; cold medications (CM) were not a risk factor for SOCs. The explanation for the disparity between the present study and the aforementioned Japanese studies might be the difference in rational drug use, which cold remedies are used less often in our country. Genetic diversity may also play an important role. Moreover, the criteria for identifying causative drugs differed between studies. Although Thailand and Japan are both located in East Asia, associations between HLA genotype and cold medicine-related SJS/TEN with SOCs in Thailand have been shown to differ from Japanese patients^34^. Besides, the incidence of CM-related SJS/TEN in our study was relatively low (n = 1, 2%); therefore, our sample size might have been inadequate to obtain statistical significance**.**

Similar to earlier reports, we did not find an association between SCORTEN and SOCs^7,29^. SCORTEN tends to use clinical parameters that reflect overall patients’ systemic conditions to predict the severity and morbidity. Since the ocular surface has a unique and privileged immune response, the discordance between ocular and systemic manifestations might explain why this score was not directly related to the severity of ocular manifestations in acute SJS/TEN.

Different inflammatory mediators and cell types have been proposed to regulate the immunopathology of SJS/TEN. In our study, S100A8/A9 levels were upregulated in patients with SJS/TEN compared to those in the control group and tended to increase in patients with severe ocular involvement. The S100A8/A9 complex is a critical alarmin that is upregulated in numerous inflammatory diseases such as rheumatoid arthritis, chronic inflammatory bowel disease, psoriasis, systemic lupus erythematosus, and atopic dermatitis^35–37^. Currently, no data are available on the potential function of S100A8/A9 in SJS/TEN. S100A8/A9 is expressed in monocytes, which are present in the epidermis of SJS/TEN skin lesions^38^, and can also be produced by epidermal keratinocytes^39^. While previous studies have implicated a role for S100A8 and S100A9 in causing accelerated inflammation^40,41^, opposite effects were reported in models of type IV-c hypersensitivity reaction^42^. S100A8/A9 inhibits dendritic cell maturation and antigen-presenting ability, leading to decreased T-cell activation and reduced intensity of immune responses in contact dermatitis^42^. Due to contradictory evidence on whether S100A8/A9 amplifies inflammation or exhibits an anti-inflammatory effect, the role of S100A8/A9 expression in the pathogenesis of SJS/TEN needs to be further elucidated.

S100A8 and S100A9 are found to be upregulated in the epidermis of the skin during the active epidermal regeneration process^43^, and S100A8/A9 could alter the skin barrier proteins in atopic dermatitis^36^. In the present study, the elevation of serum levels of S100A8/A9 possibly arose from epithelial damage to the skin in SJS/TEN. Further studies are required to investigate S100A8/A9 expression and function in SJS/TEN skin lesions.

In the eye, S100A8/A9 enhances inflammation in the ocular surface by promoting PMN infiltration and the expression of IL-1b, IL-6, and TNF-α, as an inflammatory amplifier^44,45^. Moreover, there is evidence that elevated serum S100A8/A9 levels correlate with clinically active joint and eye inflammation in autoimmune uveitis^46^. As primary ocular manifestations in acute SJS/TEN are intense ocular inflammation and epithelial sloughing, S100A8/A9 probably plays a role in ocular inflammation during the acute phase of SJS/TEN.

Our study demonstrated the elevation of several mediators in the serum of patients with SJS/TEN compared to those in HC. These results confirm the previous finding that IP-10 and IL-6 levels were elevated in the serum of patients with SJS/TEN^21,38,47,48^. IL-6 levels were also found to be higher in tears from SJS/TEN patients, although no correlation with the severity of ocular involvement was observed^20,22,49^. Studies have demonstrated that GLYN is probably a key mediator in keratinocyte apoptosis in SJS/TEN, and corneal epithelium could produce GYLN in the presence of TNF-α in an ex vivo report^25,26^. However, the fact that GYLN levels were marginally significantly higher in those with SOCs compared to those with non-severe reactions in our study might be due to the limited sample size, or the roles of GYLN in SOCs may be, in fact, not as important as that of S100A8/A9 in SJS/TEN. The functions of PDGFs in the pathogenesis of SJS/TEN remain to be explored.

After physicians confirmed the diagnosis of SJS/TEN, all patients were registered with the ThaiSCAR. This systematic registration not only provides better holistic care for patients but also helps physicians to collect comprehensive data for each individual. This study identified predictive factors for SOCs in SJS/TEN patients and found that BSA and older age were correlated with SOCs. In addition, we analyzed the correlation between inflammatory cytokines and SOCs. Previous studies have examined local specimens, including tears and conjunctival swabs, but because of the complexity involved in collecting local specimens and the lack of a standard protocol for handling samples, we decided to interpret biomarkers from serum. We found that S100A8/A9 and GYLN levels tended to increase in the SOC group. To our knowledge, this is the first report indicating that S100A8/A9 is upregulated and might be involved in the pathogenesis of SJS/TEN.

This study had a few limitations, including its retrospective design. Second, the sample size was limited due to the rarity of the disease, with a relatively small number of patients being treated at one center. Finally, all identified causes of SJS/TEN in the ThaiSCAR registry were medications; infections or other unidentified factors that could be potential etiologies of SJS/TEN causing SOCs were not included. Future large-scale, multicenter cohort studies, and the analysis of tear biomarkers or impression cytology evaluation of conjunctiva with their temporary changes are needed to verify the risk factors associated with SOCs in SJS/TEN.

In summary, these identified factors consisting of clinical manifestations and upregulated biomarkers would be beneficial for developing screening protocols or universal tools that will help physicians recognize high-risk patients, leading to prompt management and prevention of chronic severe ocular sequelae.

## Conclusion

From the clinical factor analysis, disease severity (BSA detachment ≥ 10%) and older age were predictive factors for acute SOCs in SJS/TEN. From the serum biomarker analysis, the levels of S100A8/A9, IP-10, IL-6, PDGF-AA, and PDGF-BB were increased in the SJS/TEN group; they may be potential markers that could differentiate between HC and those with SJS/TEN. The S100A8/A9 and GYLN levels tended to increase in the severe ocular involvement group, suggesting that S100A8/A9 and GYLN are involved in the pathogenesis of SOCs and serve as helpful biomarkers to predict acute SOCs in SJS/TEN.

Taken together, two clinical factors, namely BSA and older age, and increased levels of serum biomarkers, namely S100A8/A9 and GYLN, may guide clinicians in identifying high-risk groups that could develop acute SOCs. Prompt management in these patients will minimize the chance of developing chronic ocular sequelae, leading to vision loss.

## Methods

### Subjects and study design

This study followed the tenets of the Declaration of Helsinki and was approved by the Institutional Review Ethics Committee of the Faculty of Medicine, Chulalongkorn University. This cross-sectional study was conducted at King Chulalongkorn Memorial Hospital (KCMH), Bangkok, Thailand, between July 2020 and January 2021. Forty-seven patients diagnosed with SJS/TEN at KCMH were enrolled in this study. These patients were part of the Thailand Severe Cutaneous Adverse Reactions (ThaiSCAR) cohort registered at ClinicalTrials.gov (NCT02574988). The study protocol was approved by the Institutional Review Board of the Faculty of Medicine, Chulalongkorn University (approval no. 1272/2020, IRB No. 454/63). Informed consent was obtained from all participants. The classification criteria of Bastuji-Garin et al. were used to describe SJS, TEN, and SJS/TEN overlap syndrome^1^ and the suspected culprit drugs were assessed according to the algorithm of drug causality for epidermal necrolysis (ALDEN)^50^.

According to the ThaiSCAR protocol, SJS/TEN patients were admitted after the onset of symptoms. Patients’ serum was then collected, and referrals were made to the ophthalmologist. The ocular complications were evaluated primarily within 24 h of admission.

### Demographic and clinical data

We retrospectively reviewed the medical records and electronic database of patients with SJS/TEN. The inclusion criteria were as follows: (1) patients with fully accessible medical records of the acute phase for evaluation of prognostic factors (2) no history of other ocular surface disorders or ocular surgeries, and (3) age > 18 years. For simplicity, patients with SJS/TEN overlap were assigned to the TEN group.

The clinical parameters and ocular findings were collected during the acute phase of SJS/TEN, within 8–12 days of symptom onset^51^.When the acute ocular severity differed between the eyes, the eye with greater severity was chosen for evaluation. If both eyes were symmetrically severe, the right eye was selected. The acute ocular severity score was defined as previously described by Sotozono et al.^27^ (Table 2). Patients with ocular severity scores greater than grade 2 were considered to have SOC.

### Evaluation of clinical parameters for predicting SOC in SJS/TEN

The study population was divided into two groups according to the severity of ocular involvement: STS/TEN patients with non-SOC (acute ocular severity grade 0,1, and 2; n = 27) and SJS/TEN patients with SOC (acute ocular severity grade 3; n = 20). The disease severity (SJS: BSA detachment < 10%, SJS/TEN overlap: BSA detachment 10%–30%, TEN: BSA detachment > 30%), age, sex, laboratory results, SCORTEN, mucosal involvement, causative drugs, flu-like symptoms, underlying diseases, initial visual acuitiy, sepsis complications, and HIV infection in the acute phase were analyzed.

### Serum collection

The serum samples of patients with SJS/TEN were cryopreserved from a ThaiSCAR prospective study.

Serum (30 ml) was collected from patients registered in the ThaiSCAR database while admitted to KCMH during the acute phase of SJS/TEN, within 8–12 days of symptom onset^51^. Undiluted samples were stored at − 80 °C until biomarker measurement. Patients in this study underwent serum collection and ocular examination within a maximum of 3 days following admission.

### Evaluation of biological markers associated with SJS/TEN

Because sepsis appears to be a possible confounder, seven patients with sepsis were excluded. Serum from 40 patients with SJS/TEN and 18 HC was analyzed to explore the relationship between serum biomarkers in SJS/TEN and HC. A non-significant difference was noted in the mean age between the SJS/TEN and HC groups. After thawing, the samples were analyzed using a bead-based multiplex immunoassay. Panels of 42 biomarkers were used to identify biomarkers with prognostic potential for SJS/TEN. All samples were analyzed in duplicate. Biomarkers with a GMR of more than two were considered clinically significant.

### Subgroup analysis to determine biological markers associated with SOC in SJS/TEN

To identify the biological markers related to acute SOCs, we analyzed and compared the data between SJS/TEN patients with non-SOC (n = 24) and SOC (n = 16).

### Multiplex soluble analysis

To quantify soluble analytes simultaneously in plasma samples, we do the Human Th17 Cytokine Panel 7-plex (IL-6, IL-10, IFN-γ, TNF-α, Th17A, Th17F and Th22), the Human Growth Factor Panel 13-plex (Angiopoietin-2 (Ang-2), EGF, EPO, FGF-basic, G-CSF, GM-CSF, HGF, M-CSF, PDGF-AA, PDGF-BB, SCF, TGF-α and VEGF), Human Vascular Inflammation Panel 13-plex (Myoglobin, Calprotectin (S100A8/A9), Lipocalin A (NGAL), C-Reactive Protein (CRP), MMP-2, Osteopontin (OPN), Myeloperoxidase (MPO), Serum Amyloid A (SAA), IGFBP-4, ICAM-1 (CD54), VCAM-1 (CD106), MMP-9, and Cystatin C), and the custom Human panel 9-plex (TGF-b1, IL-18, IP-10, MCP-1, sFASL, IL-15, Rantes, IL-23, and Granulysin) were used. (Biolegend®, San Diego, CA, U.S.A.). 25 μl of assay buffer was added into each well. Then, 25 μl of diluted standard or plasma were added in standard or sample wells. After that, 25 μl of mixed beads and 25 μl of detection antibodies were added into each well and incubated for 2 h at room temperature on an orbital plate shaker. After incubation, 25 μl of streptavidin-PE solution was added and then incubated for 30 min at room temperature on an orbital plate shaker. The plates were centrifuged at 1,000 rpm for 5 min. After decanting liquid and wash more one time with wash buffer, all wells added 150 μl of wash buffer and shaked for 2–3 min prior to analyze by flow cytometry (BD FACSCalibur™, Becton Dickinson, USA).

### Statistical analysis

Data were analyzed as means and standard deviations for continuous variables and counts and percentages for categorical variables. Univariate and multivariate logistic regression analyses were used to investigate prognostic factors related to SOCs. Multivariate logistic regression analysis was performed by stepwise backward elimination approach with variables whose *P* value were less than 0.2 in univariate analysis. The biomarker levels were log-transformed and described as geometric means (GM) with the percent coefficient of variation (%CV). Differences between groups was presented as geometric mean ratios (GMR). Normal distribution was determined using histograms and the Shapiro–Wilk test for normality after logarithmic transformation. Linear regression analysis was used to evaluate the differences in logarithmic mean values of the biomarker concentrations among various groups (healthy controls group vs. SJS/TEN group and non-severe ocular involvement group vs. severe ocular involvement group). Mann–Whitney test was used for comparison between HC and SJS/TEN groups and between SJS/TEN with non-severe and severe ocular complications groups. Statistical significance was defined as *P* < 0.05. Scatter plot graphs were created by GraphPad Software. All analyses were performed by STATA Statistical software version 15.1 (StataCorp, LLC, College Station, Texas, USA).

## Data Availability

The data analyzed from the patient’s clinical parameters and serum biomarkers that support the results of this study is available upon reasonable request from the corresponding author V.P.
